# Quantifying Gut Microbial Short-Chain Fatty Acids and Their Isotopomers in Mechanistic Studies Using a Rapid, Readily Expandable LC–MS Platform

**DOI:** 10.1021/acs.analchem.3c04352

**Published:** 2024-01-30

**Authors:** Cheng-Yu Charlie Weng, Christopher Suarez, Shawn Ehlers Cheang, Garret Couture, Michael L. Goodson, Mariana Barboza, Karen M. Kalanetra, Chad F. Masarweh, David A. Mills, Helen E. Raybould, Carlito B. Lebrilla

**Affiliations:** † Department of Chemistry, University of California Davis, Davis, California 95616, United States; ‡ School of Veterinary Medicine, University of California Davis, Davis, California 95616, United States; § Department of Food Science and Technology, University of California Davis, Davis, California 95616, United States

## Abstract

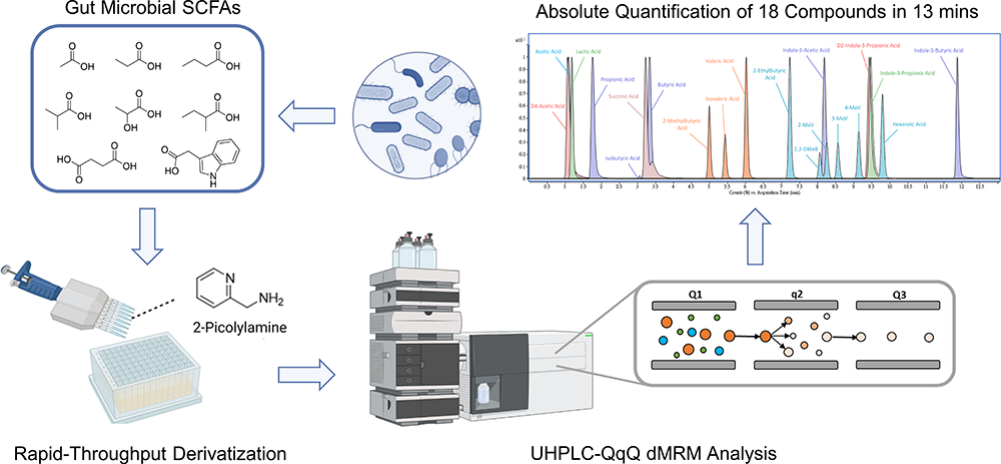

Short-chain fatty acids (SCFAs) comprise the largest group of gut microbial fermentation products. While absorption of most nutrients occurs in the small intestine, indigestible dietary components, such as fiber, reach the colon and are processed by the gut microbiome to produce a wide array of metabolites that influence host physiology. Numerous studies have implicated SCFAs as key modulators of host health, such as in regulating irritable bowel syndrome (IBS). However, robust methods are still required for their detection and quantitation to meet the demands of biological studies probing the complex interplay of the gut-host-health paradigm. In this study, a sensitive, rapid-throughput, and readily expandible UHPLC-QqQ-MS platform using 2-PA derivatization was developed for the quantitation of gut-microbially derived SCFAs, related metabolites, and isotopically labeled homologues. The utility of this platform was then demonstrated by investigating the production of SCFAs in cecal contents from mice feeding studies, human fecal bioreactors, and fecal/bacterial fermentations of isotopically labeled dietary carbohydrates. Overall, the workflow proposed in this study serves as an invaluable tool for the rapidly expanding gut-microbiome and precision nutrition research field.

## Introduction

Short-chain fatty acids (SCFAs) are the primary metabolites produced by the fermentation of indigestible dietary polysaccharides, such as fiber, by the gut microbiome.^1^ SCFAs have the potential to modulate the gut microbiome as they play key roles in host energy metabolism, health maintenance, and disease development.^1−3^ Studies have demonstrated that fecal SCFA profiles can be directly correlated to host physiology and serve as a noninvasive, reliable biomarker for various disease states.^4−7^ For example, the difference between fecal propionic and butyric acid concentrations has been used to distinguish IBS patients from healthy subjects.^8^ Furthermore, other studies have also shown that SCFAs regulate the neuro-immunoendocrine system, thereby encouraging a number of recent gut microbiota–brain axis studies.^9^ Additionally, branched short-chain fatty acids (BSCFAs) are produced in less abundant quantities in the large intestine during the fermentation of branched-chain amino acids by the gut microbiome.^10^ Although recent work has demonstrated the importance of BSCFAs to host metabolism,^11^ the relationship between BSCFAs and host health has not been fully explored. Despite the need for more mechanistic studies of gut–host interactions, progress in these areas has been limited by the lack of rapid-throughput analytical methods for the absolute quantification of SCFAs and BSCFAs, simultaneously in biological tissues such as feces and serum. Furthermore, isotopic labeling studies with mass spectrometry have become an attractive strategy for elucidating the gut microbial metabolism of indigestible dietary components.^12^ These studies require rapid, sensitive, and highly expandable methods to accommodate many analytes of interest. The lack of analytical tools has hindered the analysis of large clinical and preclinical studies.

Traditional quantitation methods for SCFAs include gas chromatography (GC), high-performance liquid chromatography (HPLC), and capillary electrophoresis (CE) coupled to detection methods such as flame ionization, UV absorption, and mass spectrometry, among others.^13^ Additionally, nuclear magnetic resonance has been used for the quantification of SCFAs. However, existing methods lack both sensitivity and speed. Novel ambient mass spectrometry methods have been developed for speed;^14^ however, liquid and GC techniques coupled with mass spectrometry remain the most commonly employed to analyze SCFAs in human serum or feces.^15,16^ The analysis of BSCFA is similarly performed but has not been widely integrated with SCFA. For sensitivity and sample stability, precolumn derivatization reagents such as 2-nitrophenylhydrazine,^17^ 3-nitrophenylhydrazine,^16^ aniline,^18^ Girard’s reagent T,^19^ and benzyl chloroformate are often used.^20^ However, many of these derivatizations require complicated and time-consuming pretreatment steps that significantly increase the workload and analysis time. More recently, the popularity of metabolomics methods has been suggested as a solution for SCFA and BSCFA; however, the lack of quantitation and the poor overlap with these classes of compounds render them ineffective for a more comprehensive analysis. Additionally, the use of stable isotopic labeling to probe the mechanism of digestion produces a potentially large number of isotopomeric species that would similarly require identification and quantitation.

In this study, we propose a rapid-throughput and simplified method for the combined analysis of SCFA, BSCFA, and isotopically labeled homologues. It employs ultrahigh-performance liquid chromatography-triple quadrupole mass spectrometry (UHPLC-QqQ MS) with 2-picolylamine derivatization.^21^ The method is fast, sensitive, and highly expandable to include additional compounds that are relevant to the microbiome, such as in mice feeding, bioreactors, and isotopic labeling studies. The method employs a 96-well plate format, which is not readily amendable to GC–MS, and effective derivatization that produces optimal LC and rapid separation (17 min run). The use of dynamic multiple reaction monitoring (dMRM) produces high sensitivity and absolute quantitation in a short-run format.

The method was then applied to various studies, including (1) the measurement of SCFA (with BSCFA) concentrations in the cecal of low-fat diet-fed and high-fat diet-fed mice, (2) the measurement of SCFAs in human fecal bioreactor studies, and (3) the mechanistic study of SCFA production in fecal and isolated bacterial strain-based fermentations using isotopically labeled dietary carbohydrates. We demonstrate the expandability of the platform by adding the BCFAs and the full series of isotopically labeled SCFA homologues according to their number of ^13^C-labeled carbons. The results from these case studies highlight the utility of the method as a tool for probing the host-gut-health paradigm.

## Experimental Section

### Materials

Methanol (MeOH) of HPLC grade, 2-picolylamine (2-PA), dipyridyl disulfide (DPDS), triphenylphosphine (TPP), acetic acid, propionic acid, butyric acid, valeric acid, caproic acid, lactic acid, succinic acid, isobutyric acid, isovaleric acid, 2-methylbutyric acid, 3,3-dimethylbutyric acid, 2-methylvaleric acid, 3-methylvaleric acid, 4-methylbutyric acid, indole-3-acetic acid, indole-3-butyric acid, indole-3-lactic acid, 2-ethylbutyric acid (2-EtB), d^4^-acetic acid, glucose, galactose, fructose, arabinose, fucose, rhamnose, glucuronic acid, galacturonic acid, Nacetylglucosamine, *N*-acetylgalactosamine, mannose, allose, ribose, 3-methyl-1-phenyl-2- pyrazoline-5-one (PMP), trifluoroacetic acid (TFA), and ammonium acetate were purchased from Sigma-Aldrich (St. Louis, MO). d^2^-indole-3-propionic acid was purchased from Toronto Research Chemicals (Toronto, Canada). Algal starch (U–^13^C, 98%+), ^13^C_6_ glucose, and unlabeled algal starch were purchased from Cambridge Isotope Laboratories (Tewksbury, MA). Isopropanol of LC/MS grade was purchased from Fisher Scientific (Waltham, MA). Acetonitrile (HPLC-grade) was purchased from Honeywell (Muskegon, MI).

### Derivatization of SCFAs in 96-Well Plates

A pooled standard solution consisting of 18 carboxylic acid metabolites was prepared in MeOH and serially diluted to different concentrations ranging from 0.001 to 500 μg/mL based on their abundances in the samples of interest, where the range of each analyte could be found in Table 1. An internal standard mixture containing 100 μg/mL of d^4^-acetic acid, 50 μg/mL of d^2^-indolepropionic acid, and 10 μg/mL of 2-ethylbutyric acid was spiked into all standards and samples at a ratio of 1:10 (v/v). 200 μL of ACN and 100 μL of derivatization reagent containing 20 mM TPP, 20 mM DPDS, and 20 mM 2-PA were plated in a 1 mL 96-well plate beforehand. A 10 μL aliquot of standard/sample was added, the plate sealed, and the sample was incubated at 60 °C for 10 min. The whole procedure was completed in a 4 °C cold room to reduce volatile analyte evaporation. After the reaction was complete, the derivatized samples were dried in a miVac concentrator (SP Industries, Warminster, PA). The dried samples were reconstituted in 500 μL of 50% MeOH before instrumental analysis.

**Table 1:** Quantitative Information on Derivatized SCFAs

compound	RT (mins)	precursor[M + H]^+^ (*m*/*z*)	quantifier/qualifier (*m*/*z*)	optimized CE	cal curve range (ug/mL)	internal standard
acetic acid	1.15	151	109/133	14/10	5–500	D4-AA
lactic acid	1.28	181	109/92	15/23	2–250	D4-AA
propionic acid	1.85	165	109/92	15/23	5–500	D4-AA
isobutyric acid	3.11	179	109/161	15/11	0.05–2.5	2-ETB
succinic acid	3.26	299	191/109	16/12	0.8–25	2-ETB
butyric acid	3.32	179	109/92	15/23	5–500	D4-AA
2-methylbutyric acid	5.03	193	109/175	15/11	0.05–2.5	2-ETB
isovaleric acid	5.50	193	109/175	15/11	0.05–2.5	2-ETB
valeric acid	6.08	193	109/175	15/11	0.16–10	2-ETB
2,2-dimethylbutyric acid	8.10	207	109/92	16/28	0.002–0.1	2-ETB
indole-3-acetic acid	8.18	266	109/NA	24/NA	0.004–0.5	D2-IPA
indole-3-lactic acid	8.23	296	109/NA	20/NA	0.004–0.5	D2-IPA
2-methylvaleric acid	8.25	207	109/NA	15/NA	0.002–0.1	2-ETB
3-methylvaleric acid	8.59	207	109/NA	15/NA	0.002–0.1	2-ETB
4-methylvaleric acid	9.20	207	109/NA	15/NA	0.002–0.1	2-ETB
indole-3-propionicic acid	9.51	280	201/109	28/20	0.002–0.1	D2-IPA
caproic acid	9.88	207	109/99	15/15	0.001–1	2-ETB
indole-3-butyric acid	11.87	294	109/NA	16/NA	0.002–0.1	D2-IPA
D4-acetic acid (D4-AA)	1.11	154	110/92	14/18		
2-ethylbutyric acid (2-ETB)	7.26	207	109/71	15/19		
D2-indole-3-propionic acid (D2-IPA)	9.45	282	130/110	28/20		

### LC–MS/MS Analysis

Derivatized samples were analyzed on an Agilent 6495B QqQ MS coupled to an Agilent 1290 Infinity II UHPLC. Separation was performed on an Agilent Poroshell 120 EC-C18 column (2.1 × 100 mm, 1.9 μm particle size). Aqueous mobile phase A consisted of 100% nanopure water. Organic mobile phase B consisted of a 1:1 (v/v) ACN/IPA mixture. The following binary gradient was used: 0.00–1.00 min, 5.00% B; 1.00–10.00 min, 5.00–20.00% B; 10.00–11.00 min, 20.00% B; 11.00–15.00 min, 20.00–60.00% B; and 15.00–16.00 min, 60.00–5.00% B. 1 μL portion of the sample was injected into each run. The mobile phase flow rate was 0.45 mL/min, and the column temperature was set to 45 °C. The Jet Stream Technology (AJS) electrospray ionization (ESI) ion source was operated in the positive ion mode with the following parameters: capillary voltage = 1800 V, nozzle voltage = 1500 V, gas temperature = 240 °C, gas flow = 20 L/min, nebulizer = 25 psi, sheath gas temperature = 300 °C, and sheath gas flow = 9 L/min. Mass spectrometry data was collected in the dMRM mode.

### Method Validation

All the method validations followed revised FDA guidelines.^22^ Method reproducibility (precision) was assessed by pooling 5 random cecal samples together. The pooled sample was submitted to the derivatization workflow and instrumental analysis in six replicates. The coefficient of variation (CV) was chosen as the indicator of reproducibility and was calculated as the ratio of the standard deviation (σ) to the mean (μ). The accuracy and matrix effect were evaluated by the recovery test. The pooled samples mentioned above were spiked with a known level of SCFA standards. The recovery rate was calculated by dividing the experimental concentrations by the calculated concentrations after spiking. The limit of detection (LOD) was estimated via blank samples because of the high background interference of acetate and was calculated based on a published guideline.^23^ In brief, LOD equals 3.9 times the standard deviation of the blank (pseudoblank) signals and is then divided by the slope of the calibration curve.

### SCFA Levels in the Cecal Content of High-Fat Diet Mice and Low-Fat Diet Mice

All animal experiments were approved by the Institutional Care and Use Committee (IACUC) of the University of California, Davis. Mice were fed *ad libidum* (except as noted for specific experimental procedures) and housed on a 12:12 h light–dark cycle. Upon arrival in the facility, C57BL/6J male mice (8 week old, The Jackson Laboratory, Sacramento, CA room RB07) were immediately individually housed and fed a low-fat control diet to acclimate to the vivarium. After 1 week, mice were counterbalanced by body weight and assigned to either a modified AIN-93G low-fat (LF; 10% kcal from fat) or high-fat (HF; 45% kcal from fat) diet intervention. A detailed diet composition is provided in Table S1. After 8 weeks on diet, mice were euthanized by pentobarbital overdose (Fatal Plus, Vortech Pharmaceuticals, Dearborn, M I; 300 mg/kg; i.p.) and exsanguinated by cardiocentesis. Cecal contents were isolated in pretared 2 mL screw cap tubes with O-ring seals (Sarstedt, Nümbrecht, Germany; Cat. no. 72.694.396) and immediately frozen on dry ice. Cecal content samples were stored at −80 °C prior to analysis. To extract SCFAs from cecal contents, a 50 mg/mL solution was prepared in 70% MeOH, homogenized for 5 min by vortexing, and centrifuged at 13,500 rpm for 10 min at 4 °C. The supernatant was then subjected to derivatization.

### SCFA Production in Fecal Bioreactors

Batch fecal fermentations were conducted in triplicate using stool from 5 adult subjects. All procedures involving the use of human fecal samples were approved by The University of California Davis Institutional Review Board (IRB #1600677). To prepare the fecal inoculum, frozen feces was thawed in an anaerobic chamber on ice. 4 g of feces was weighed into a 50 mL centrifuge tube containing 6 mL of sterile, deoxygenated 1.67× PBS, 33% v/v glycerol, then vortexed at full speed for 5 min. The slurry was centrifuged for 5 min at 200*g* at 4 °C to settle nonmicrobial solids. In the anaerobic chamber, the supernatant was transferred into 15 mL centrifuge tubes and frozen at −80 °C until needed. The composition of the fermentation medium was based on that of Walker et al.^24^ with the following changes to the buffer composition: 5 g/L K_2_HPO_4_, 3.19 g/L KH_2_PO_4_, 1.35 g/L NaHCO_3_, 1.63 g/L Na_2_CO_3_, and 9.76 g/L MES•H_2_O. Homogenized feces were thawed on ice, vortexed, and then centrifuged at 200*g* at 4 °C for 5 min. An aliquot of 1 mL of fecal slurry supernatant was added to 15 mL of fermentation media in prepared sterile fermentation tubes. Fermentations were carried out in triplicate at 37 °C in a Coy anaerobic chamber for each of the 5 subjects. Samples for SCFA analysis were collected on day 3 and stored at −80 °C. After centrifugation at 13,500 rpm for 10 min at 4 °C, the supernatant was directly used for derivatization.

### Fecal and Bifidobacterium Fermentations with ^13^C Starch and ^13^C Glucose

A 1% w/v solution of each substrate was 1%-inoculated with *Bifidobacterium pseudocatenulatum* MP80 and a fecal slurry from an anonymous human donor, respectively, and incubated for 36 h at 37 °C in a Coy anaerobic bubble with an atmosphere of 3% hydrogen, 5% CO2, and balance nitrogen. The feces fermentation was in a fermentation medium with the buffer solution modified as follows: K_2_HPO_4_, 5 g; KH_2_PO_4_, 3.19 g; NaHCO_3_, 1.35 g; Na_2_CO_3_, 1.63 g; MES•H_2_O, and 9.76 g per liter.^24^ Additionally, no background polysaccharides were added. The fecal slurry was a 10% w/v suspension of feces in 1x anaerobic PBS. The pure culture fermentation was in modified MRS broth without glucose,^24^ and the inoculum was grown overnight in MRS broth +0.05% l-cysteine HCl. 1 mL of each batch fermentation was collected at 0 and 36 h and stored at −80 °C. After centrifugation at 13,500 rpm for 10 min at 4 °C, the supernatant was subjected to derivatization.

### Quantitation of Total Monosaccharides in Fecal and Bifidobacterium Fermentations

The monosaccharide analysis of the fermentation media was adapted from Amicucci et al.^25^ In brief, 10 μL aliquots from homogenized stock solutions were transferred to a 96-well plate (2 mL wells). Each sample was subjected to hard acid hydrolysis (4 M trifluoracetic acid for 1 h at 121 °C), after which the reaction was quenched by the addition of 855 μL of nanopure water. Following hydrolysis, 10 μL aliquots of the hydrolyzed sample and 50 μL of an external calibration curve of 14 monosaccharides with concentrations ranging from 0.001–100 μg/mL were derivatized with 0.2 M 1-phenyl-3-methyl-5-pyrazolone (PMP) in 1:1 methanol and 28% NH_4_OH for 30 min at 70 °C. After completion of the reaction, derivatized samples were dried overnight by vacuum centrifugation, reconstituted in nanopure water, and excess PMP was removed by chloroform extraction. A 1 μL aliquot of the aqueous layer was injected into an Agilent 1290 Infinity II UHPLC system. Separation was achieved using an Agilent Poroshell HPH-C18 column (2.1 × 50 mm, 1.9 μm) and guard in 2 min with an isocratic elution of 12% solvent B. Solvent A consisted of 25 mM ammonium acetate adjusted to pH 8.2 using a concentrated ammonia solution, and solvent B consisted of 95% acetonitrile in water. The separated glycosides were then detected by an Agilent 6495A QqQ-MS operated in multiple reaction monitoring (MRM) mode, and quantitation of monosaccharides was achieved by comparison to the external calibration curve.

## Result and Discussion

### Rapid-Throughput and Expandable Derivatization Methods

**Figure 1:**
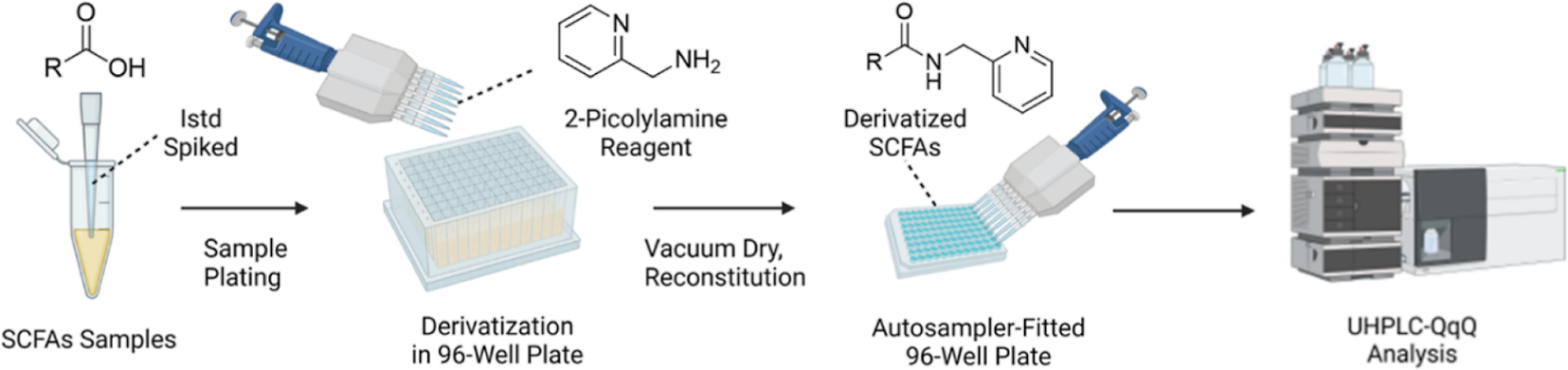
Schematic of the rapid-throughput SCFA quantification platform.

We introduce a rapid-throughput platform to quantify up to 18 common gut microbe-derived metabolites with carboxylic groups in a 17 min LC–MS run. The overall workflow is summarized in Figure 1. Samples were spiked with internal standards (IS) before plating to mitigate matrix effects, increase reproducibility, and achieve absolute quantification. For the internal standards, three compounds were used to represent all the compounds, as many were not commercially available. Thus, shorter carbon chain SCFAs (less than 4 carbons) were corrected with d^4^-acetic acid, longer chain SCFAs and BSCFAs with 2-ETB, and indole derivatives were corrected with d^2^-indolepropionic acid according to the similarity of their chemical structure and physical properties. The derivatization protocol was adjusted from a 2-picolylamine method described previously to accommodate rapid-throughput analysis in the 96 well plate format, representing a significant advancement.^21^ Derivatization reagents containing 20 mM 2-picolylamine, 20 mM TPP, and 20 mM DPDS were premixed into a single reagent solution. Then, 100 μL of the reagent, 200 μL of ACN, and a 10 μL aliquot of each sample were added to each well of a 96-well plate using a multichannel pipet. After the reaction and solvent evaporation, the reconstitution and transfer steps were also carried out with multichannel pipettes to save labor and further reduce sample preparation time.

### LC–MS/MS Analysis

**Figure 2:**
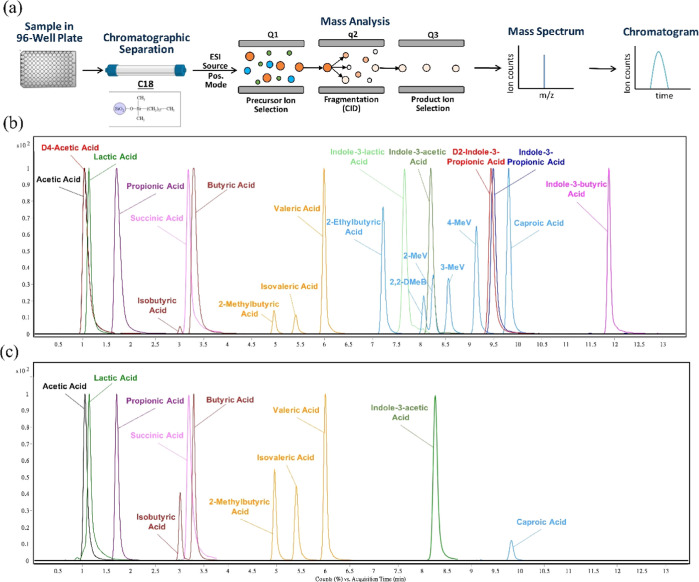
(A) Instrumental analysis workflow. (B) Chromatogram of standards and (C) bioreactor fermentation samples. To facilitate comparison across chromatograms, we scaled each chromatogram by the highest signal in the corresponding MRM transitions.

The overall instrumental analysis workflow is shown in Figure 2a. Derivatized SCFAs were submitted to LC separation after injection by the autosampler. Chromatography (shown in Figure 2b,c) was optimized to reach the base peak separation of structural isomers that have the same MRM transitions. A C18 column was used, and the organic phase was 1:1 (v/v) ACN/IPA. The analyte with the longest chain (six carbons) had the highest retention time (RT) at around 12 min. The total run time per sample was 17 min, including column re-equilibration, which facilitated the analysis of large sample sets.

After elution, analytes were ionized via an ESI source operated in the positive ion mode. Ion source parameters were optimized using the Agilent Source Optimizer. Precursor ion masses (protonated) were determined by an MS1 scan, and the top two MS2 fragments for each analyte were chosen as the quantifier and the qualifier transitions for MRM, respectively. Optimized collision energies (CEs) were also obtained for each transition by using Agilent Optimizer software. The RT, MRM transitions, and optimal CEs used for different analytes are reported in Table 1.

An external calibration curve was built and applied to achieve absolute quantification. Standards of all analytes were pooled together in different concentrations and serially diluted based on their abundance in test samples, and internal standards were spiked in the calibration curves. The ratio of analyte signals to internal standard signals was used for quantification, increasing the reproducibility and enhancing the dynamic range of the calibration curves. The concentration range of calibration curves and the internal standard applied for each analyte are shown in Table 1.

To evaluate the matrix effect in target samples and the robustness of this method, we performed an analysis on pooled cecal samples. Acetic acid showed the largest variability due to its volatility and large background because of its ubiquity in the environment. We consistently observed a high acetic acid background signal, which reduced its LOD. To solve this issue, we implemented a background correction using isotopically labeled acetic acid by subtracting any observed signal in the blank from that of the sample. To ensure consistency, all other analytes were also background-corrected in this manner. All analytes showed good linearity in their respective calibration curves (Figure S1). The LOD of acetic acid is higher than other analytes due to the background issue and a lower ionization efficiency, but the LOD is still much lower than the normal concentrations in the biological samples, demonstrating excellent sensitivity when compared to traditional methods. The accuracy of the method was tested by a spike-in recovery test. A known concentration of standards was spiked into the pooled cecal sample. The recovery rate for the abundant SCFAs (e.g., acetate, lactate, propionate, and butyrate) in the matrix was within 10% of the nominal concentration. Reproducibility was also assessed on the pooled cecal sample. A CV value of less than 10% was obtained for nearly all analytes. All method validation results are summarized in Table S2.

### Determination of SCFA in the Cecal Content of High-Fat-Diet and Low-Fat-Diet Mice

To demonstrate the utility of this method, we first analyzed cecal samples from mice. Previous studies have demonstrated that high-fat diets can alter the gut microbiota and host metabolism, resulting in chronic health issues.^26^ Therefore, analyzing SCFA levels in the cecal content is a promising approach for identifying and monitoring changes in the gut microbiota caused by dietary interventions or other factors.^27,28^ To evaluate the influence of a high-fat diet on the production of SCFAs, we analyzed the SCFA levels in cecal samples from adult C57BL/6J mice fed a high-fat diet (*n* = 17) and a low-fat diet (*n* = 10).

**Figure 3:**
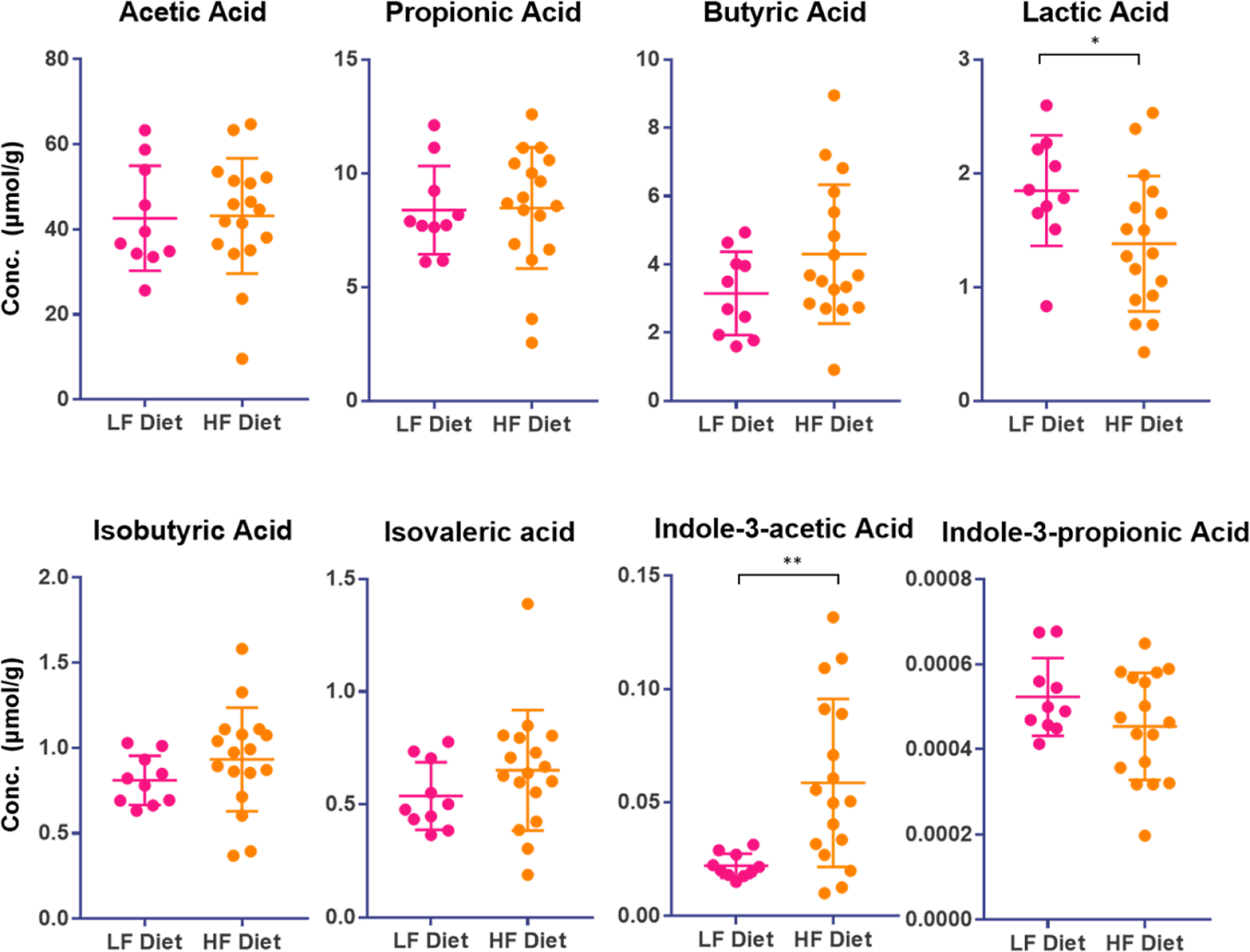
Absolute quantification results of cecal SCFA concentrations in high-fat-diet mice (*n* = 17) and low-fat-diet mice (*n* = 10). Significant differences were determined by Student’s *t*-test. (*, *p* < 0.05; **, *p* < 0.01).

The results of eight selected carboxylic acid metabolites are shown in Figure 3. The results from all carboxylic acid metabolites measured are listed in Table S3. Differences between the two groups were observed in lactic acid and indole-3-acetic acid, where lactic acid was higher in the low-fat diet control group, while indole-3-acetic acid was higher in the high-fat diet group. Past studies have shown that high-fat diet-induced oxidative stress leads to strain selection and misbalance in *Lactobacillus*,^29^ which is one of the major lactic acid-producing bacteria genera in the mammalian intestine. The lower production of lactic acid in the high-fat diet group may have been caused by decreases in *Lactobacillus* in the intestine. Endogenous indole-3-acetic acid is mostly metabolized from dietary tryptophan via different pathways, such as the indole-3-acetamide and tryptamine pathways.^30^ Our analysis revealed a higher concentration of indole-3-acetic acid in the high-fat diet group compared to the control group, which suggested alterations in the gut microbiota of the former. The increased abundance of microbiota with enzymes capable of carrying out the metabolism pathways of indole-3-acetic acid could be responsible for this variation. Additionally, we observed that the standard deviations for most of the carboxylic metabolites were higher in the high-fat diet group than in the control group. This may be attributed to the perturbation of the gut microbiota induced by the high-fat diet. These findings highlight the potential impact of dietary interventions on the gut microbiome and its metabolism, which could be further explored to develop targeted interventions for metabolic disorders.

### SCFA in the Bioreactors Fermenting Fecal Microbiome from Different Subjects

Recent studies have highlighted the crucial role of the diet in shaping the gut microbiome. By selectively modulating the abundance of specific bacterial genera, dietary interventions have emerged as a potential approach for treating dysbiosis-related diseases.^31^ Despite significant progress in elucidating the complexity of gut microbial populations, a more comprehensive understanding is needed of the metabolic products of these microorganisms when they are treated with different substrates. However, human intervention studies pose significant challenges due to the presence of numerous confounding factors that are difficult to control or standardize, including the environment, background diet, and lifestyle. These extraneous factors can significantly impact the gut microbiome, potentially leading to inconclusive or misleading study results.^32^

**Figure 4:**
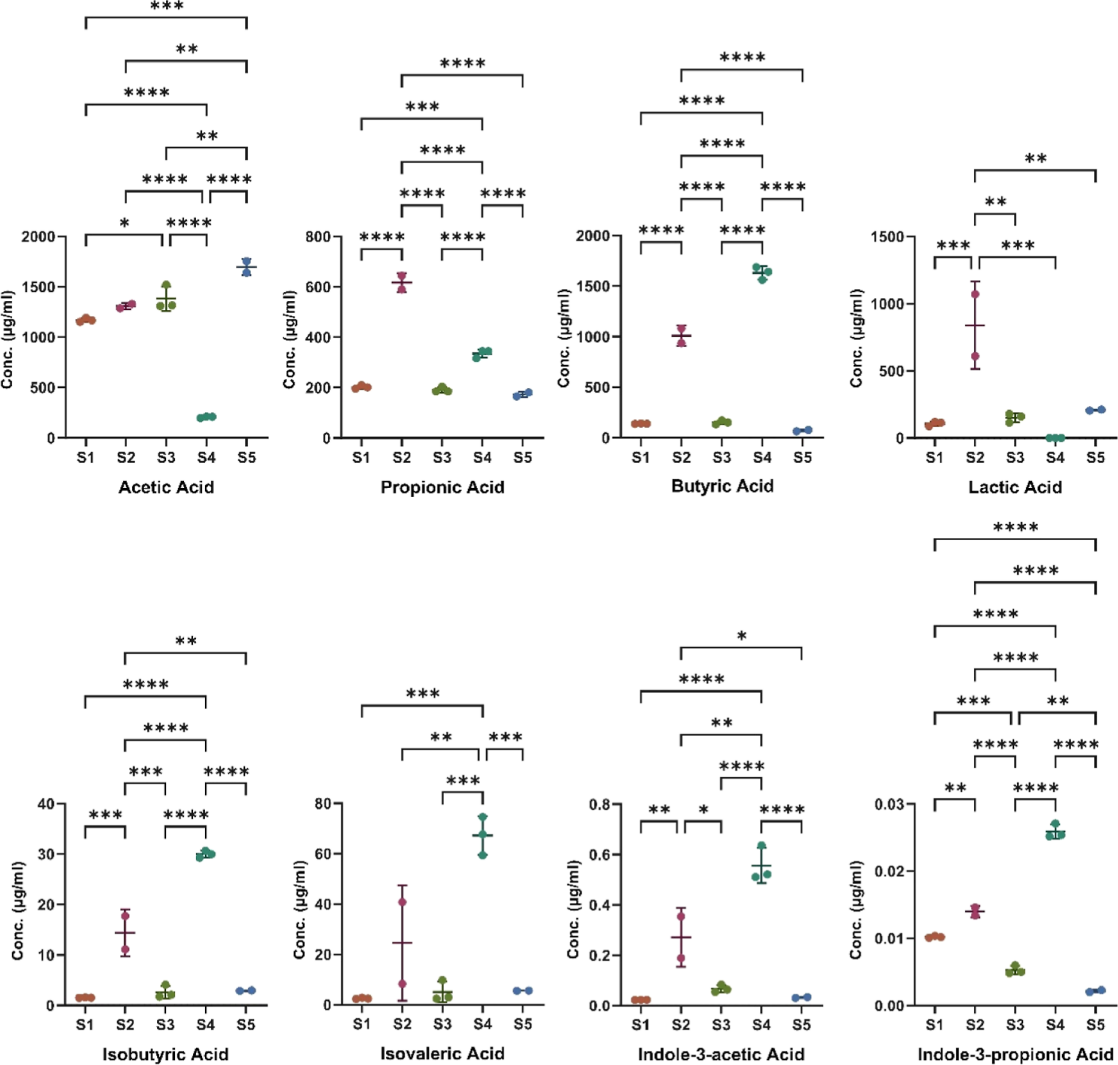
SCFA production in bioreactors fermenting the feces of five different subjects after 3 days (*n* = 3). The results showed variations in the levels of SCFAs produced among the subjects, indicating individual differences in the gut microbiota and their active metabolic pathways. The statistical significance between subjects was calculated by a one-way ANOVA followed by Tukey’s test. (*, *p* < 0.05; **, *p* < 0.01; ***, *p* < 0.001; and ****, *p* < 0.0001).

Bioreactors have become valuable tools for investigating the gut microbiome. By enabling the construction of complex gut-microbial communities in vitro, bioreactors can closely mimic the physiological conditions of the human gastrointestinal tract. As a result, bioreactor models offer a powerful solution for studying the effects of dietary interventions on the gut microbiome while minimizing interference from confounding factors.^33^ In this study, we demonstrated the applicability of our platform by measuring the SCFA production in the bioreactor-fermented feces of five different subjects over a period of 3 days. The results of the four most common SCFAs, two BSCFAs, and two indole derivatives after 3 days from biological triplicates are shown in Figure 4. All the other carboxylic acid metabolite data can be found in Table S4. Two samples with large systematic errors were removed by the Q-test. Our results reveal significant interindividual variability in the metabolic profiles of five different subjects, highlighting the diversity of gut microbiome compositions across individuals.

The bioreactor model not only has the potential to investigate the effects of specific foods on gut microbial metabolism but also could serve as a tool for phenotyping subjects based on the metabolic output of their microbiomes. For example, subjects 1, 3, and 5 exhibited similar production of propionic acid, butyric acid, lactic acid, and BSCFAs, suggesting a predominance of the same microbial metabolic pathways as compared to subjects 2 and 4. Overall, the bioreactor model paves a promising path for exploring the interplay among diet, the gut microbiome, and human health. Nevertheless, residual nutrients in fecal samples could potentially affect these results; thus, we explored isotopic labeling as a potential avenue for improving the robustness of our conclusions.

### Tracing Isotopically Labeled SCFAs in Fecal/Bifidobacterium Fermentations of ^13^C Dietary Carbohydrates

While much work has been done in correlating the gut microbial metabolism of indigestible dietary components to host physiology, much remains unknown about the full scope of metabolites produced and metabolic pathways involved. In particular, contributions from host metabolism in vivo and, in the case of fecal bioreactor studies, background nutrients found in feces act as cofounding variables in dietary intervention studies.^34^ Determining which microbial metabolic products are derived from the dietary intervention is critical to deciphering the relationship between the host and microbe. Recent studies have leveraged isotopically labeled nutrients and mass spectrometry-based methods to trace gut microbial metabolism.^35^ Whereas most studies have employed metabolomic-based methods that do not provide absolute quantitation, in this study, we highlight the utility of our targeted quantitative platform in conducting mechanistic studies of SCFA production in fermentations of ^13^C dietary carbohydrates.

**Figure 5:**
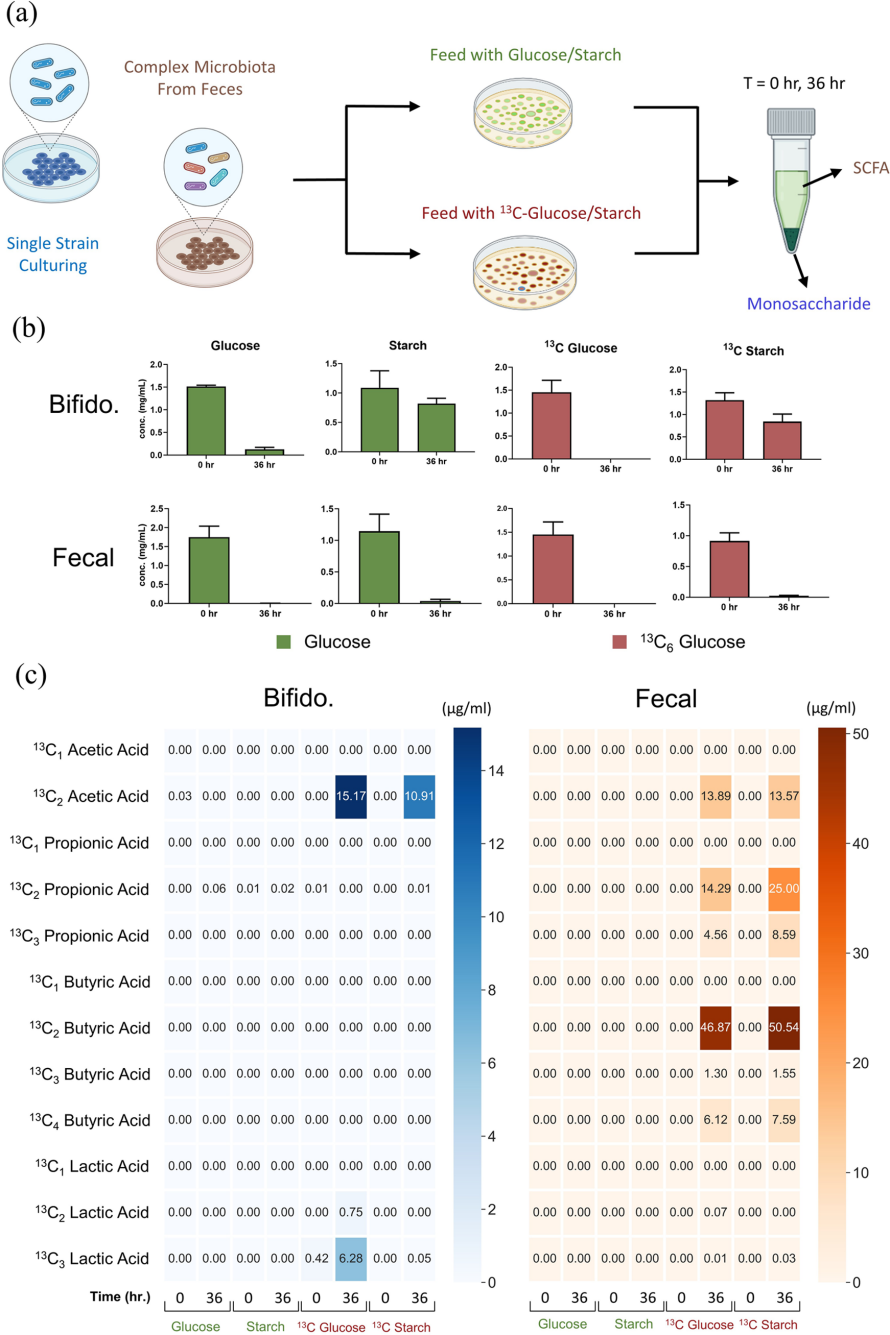
(A) Experimental design of isotope-labeled fermentations. Unlabeled and labeled glucose/starch were fermented by either a single strain of *Bifidobacterium* or a complex microbiome in human feces. (B) Glucose/^13^C_6_ glucose quantification results before and after 36 h of fermentation after performing acid hydrolysis on each supernatant. (C) Quantification results of isotopically labeled SCFAs.

Batch fermentations were conducted using both human feces, complementing the bioreactor model, and isolated *B. pseudocatenulatum*, a well-studied, beneficial member of the human gut microbiota, particularly relevant to infants. Fully labeled ^13^C glucose and ^13^C starch were selected as substrates due to their high fermentability. The consumption of the ^13^C sugars as well as their unlabeled controls is reported in Figure 5. Data for the negative controls with no inoculum are reported in Table S5. Each substrate was consumed fully by the complex microbiota found in the feces, whereas *Bifidobacterium* showed a preference for glucose and fermented starch to a lesser extent due to its large polysaccharide structure. After the inputs for microbial fermentation were determined, the isotopically labeled SCFA products were quantified, as shown in Figure 5. MRM transitions were created for each possible configuration of ^13^C labeling for the SCFAs and BCFAs, reflecting the highly expandable nature of the method. All ^13^C analyte concentrations were corrected by subtracting the isotopic distribution of the monoisotopic mass; both the uncorrected and corrected data are found in Tables S6 and S7.

The most abundant products produced by *Bifidobacterium* upon fermenting ^13^C glucose were found to be ^13^C_2_ acetate and ^13^C_3_ lactate, which agrees with the reported bifidobacteria hexose catabolism pathway, in which primarily acetate and lactate are produced in an approximate 1.5:1 molar ratio.^36^ Interestingly, no significant lactate content was observed with starch as a growth substrate in both the isotopically labeled and unlabeled controls. The results from the isolated bacterial strain culturing highlight the platform as a mechanistic probe in metabolic pathway studies.

A greater variety of isotopically labeled SCFAs was observed in the fecal fermentations, signifying a more diverse microbial community. All ^13^C sugars were fermented to completion, resulting mainly in the production of ^13^C acetate, propionate, and butyrate. However, since there are still background nutrients found in the feces, unlabeled SCFAs were still observed for fecal fermentations conducted with isotopically labeled substrates. This was further reflected in that ^13^C_2_ propionate and ^13^C_2_ butyrate were the most abundant homologues observed. The butyrl-CoA:acetate CoA transferase route involves the production of butyrate from two moieties of acetyl-CoA.^37^ We hypothesize that ^13^C_2_ butyrate is synthesized from labeled acetyl-CoA originating from the ^13^C-labeled sugar and unlabeled acetyl-CoA sourced from background nutrients in the feces. Furthermore, several bacterial-dependent pathways may contribute to the observed ^13^C SCFAs. Overall, the workflow described demonstrates the utility of the platform in unambiguously assigning the inputs and outputs of microbial fermentation. More microbial metabolites could be tracked using the same workflow, such as ^13^C amino acids, which shows the great expandability of the platform.

## Conclusions

In this study, we present a rapid-throughput and versatile platform for the absolute quantification of gut microbial carboxylic metabolites, including SCFAs, BSCFAs, and indole-derivative acids. The platform employs a 96-well plate format, enabling efficient sample pretreatment and derivatization to facilitate the analysis of large sample batches in a shorter period. Moreover, it is readily adaptable to the inclusion of other metabolites containing carboxylic acid groups, as well as monitoring many isotopologues simultaneously in labeling studies. The robustness and scalability of the platform make it a valuable tool for investigating the metabolic activity of the gut microbiome and its relationship with human health. We applied this platform to three case studies, demonstrating its ability to analyze samples with complex matrices. In one instance, metabolic profile differences between mice fed a high-fat diet and those fed a low-fat diet were investigated; in another, SCFA profiles were generated for the bioreactor fermentations of various human donors; and finally, the ^13^C fermentation products of an adult fecal sample and isolated *B. pseudocatenulatum* were characterized. Our platform is unique in its sensitivity, speed, and flexibility for application in the flourishing field of gut-microbiome research, offering a highly customizable and adaptable tool for a wide range of clinical and food intervention studies. Such a tool can ultimately shed light on gut microbial metabolism and its impact on human health.

## Supporting Information Available

Calibration curves of all analytes (Figure S1); information on LF diet and HF diet (Table S1); and LOD, reproducibility, and recovery rate of the method (Table S2) (PDF)
Concentrations of SCFAs in the cecal content of high-fat diet and low-fat diet mice (Table S3); concentrations of SCFAs in the bioreactors fermenting microbiome from different subjects (Table S4); monosaccharides data of the isotopically labeled dietary carbohydrate fermentation (Table S5); SCFAs data of the isotopically labeled dietary carbohydrate fermentation before the isotopic distribution correction (Table S6); and after the correction (Table S7) (XLSX)
