# Supplementary material for: Quantifying Gut Microbial Short-Chain Fatty Acids and Their Isotopomers in Mechanistic Studies Using a Rapid, Readily Expandable LC–MS Platform
Source: Anal Chem. 2024 Jan 30;96(6):2415–24. doi: 10.1021/acs.analchem.3c04352 (PMC10867797; doi:10.1021/acs.analchem.3c04352)
Supplement: Supplementary file 1 — ac3c04352_si_001.pdf [file ac3c04352_si_001.pdf]

Supporting Information

Quantifying Gut Microbial SCFAs and their Isotopomers in Mechanistic Studies using a Rapid, Readily Expandable LC-MS Platform

Cheng-Yu (Charlie) Weng,[1] Christopher Suarez,[1] Shawn Ehlers Cheang,[1] Garret Couture,[1] Michael L. Goodson,[2] Mariana Barboza,[1,2] Karen M. Kalanetra,[3] Chad F. Masarweh[3] David A. Mills,[3] Helen E. Raybould,[2] Carlito B. Lebrilla[1*]

[1] Department of Chemistry, University of California Davis, Davis, CA 95616, USA
[2] School of Veterinary Medicine, University of California Davis, Davis, CA 95616, USA
[3] Department of Food Science and Technology, University of California Davis, Davis, CA 95616, USA

*Corresponding Author. Email: cblebrilla@ucdavis.edu

Table of Contents

Figure S1 .... **S-3**

Table S1 .... **S-4**

Table S2 .... **S-5**

Table S3 .... **In Excel**

Table S4 .... **In Excel**

Table S5 .... **In Excel**

Table S6 .... **In Excel**

Table S7 .... **In Excel**

Concentration (X-axis) Unit: μg/ml

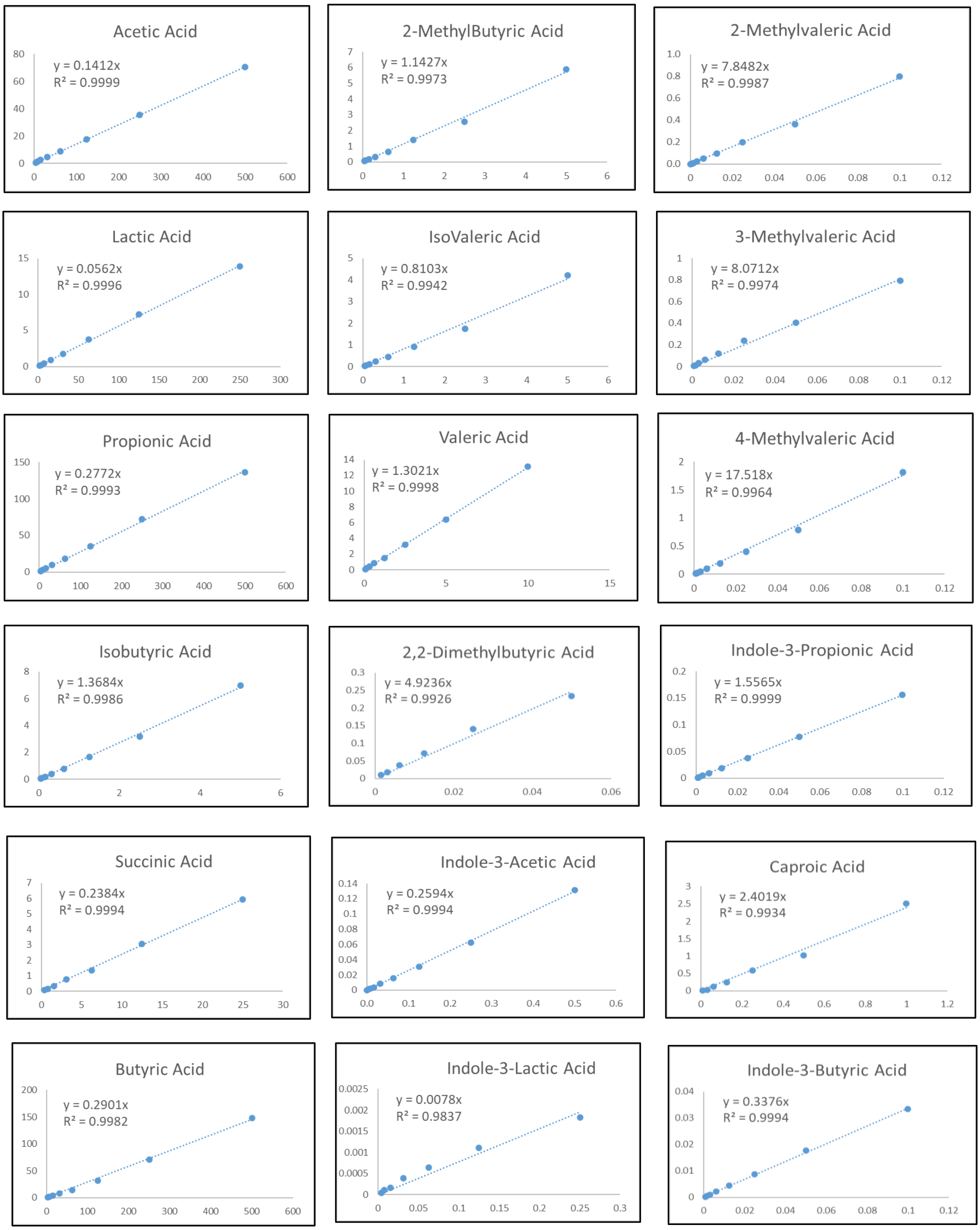

Figure S1. Calibration curves of all analytes

Table S1. Detailed diet compositions of HF diet and LF diet

	LF Diet (10% kcal fat)			***HF Diet (45% kcal fat)***			Source	Cat#
%	*gm*	*kcal*		*gm*	*kcal*			
Protein	19.5	20.0		24.4	20.0			
Carbohydrate	73.3	70.0		47.7	35.0			
Fat	4.3	10.0		24.4	45.0			
Total		100.0			100.0			
kcal/gm	3.90			4.88				
Ingredient	*gm*	*kcal*		*gm*	*kcal*			
Casein	207	826		207	826		Dyets	400601
L-Cystine	3	12		3	12		Dyets	401340
Corn Starch	535	2141		64	255		Ingredion	34010
Maltodextrin 10	125	500		125	500		Dyets	402851
Sucrose	56	223		161	642		C&H	
Cellulose	53.7	0		43.0	0		MP Bio	9004-34-6
Soybean Oil	25	225		25	225			
Lard	22	194		185	1661		Armour	
AIN-93G Mineral Mix	35	30.9		35	30.9		Dyets	210025
AIN-93 Vitamin Mix	10	39.2		10	39.2		Dyets	310025
Choline Bitartrate	2.5	0		2.5	0		Dyets	400750
TBHQ, antioxidant	0.014	0		0.014	0		Dyets	404455
FD&C Yellow Dye #5	0.04	0		0	0		Fisher	50520593
FD&C Red Dye #40	0	0		0.04	0		Fisher	50520534
FD&C Blue Dye #1	0.01	0		0	0		Fisher	50520412
Total	*1073*	*4192*		*859*	*4192*			

Table S2. Limit of detection, reproducibility (%CV) and recovery rate of the method

Compound	Method LOD (nM)	CV in Biological Samples (%)	Recovery Rate in Biological Samples (%)
Acetic acid	67.48	2.29	100.9
Lactic acid	1.85	6.83	104
Propionic acid	28.42	3.43	104.3
Isobutyric acid	28.4	6.99	112.5
Succinic acid	2.31	13.29	98.7
Butyric acid	0.65	5.68	110.3
2-Methylbutyric acid	0.04	8.77	89.3
Isovaleric acid	0.16	7.76	100.6
Valeric acid	1.2	10.1	90.4
2,2-Dimethylbutyric acid	1.58	NA	102.3
Indole-3-acetic acid	0.01	4.84	124.7
Indole-3-lactic acid	0.08	NA	118.3
2-Methylvaleric acid	0.03	8.37	93.8
3-Methylvaleric acid	0.13	5.77	93.9
4-Methylvaleric acid	0.02	7.76	86.7
Indole-3-propionicic acid	0.04	2.58	65.4
Caproic acid	2.6	7.58	88.6
Indole-3-butyric acid	0.01	3.88	120.6
